# Trait Mindfulness Is Associated With Less Amyloid, Tau, and Cognitive Decline in Individuals at Risk for Alzheimer’s Disease

**DOI:** 10.1016/j.bpsgos.2022.01.001

**Published:** 2022-01-17

**Authors:** Cherie Strikwerda-Brown, Hazal Ozlen, Alexa Pichet Binette, Marianne Chapleau, Natalie L. Marchant, John C.S. Breitner, Sylvia Villeneuve

**Affiliations:** a Centre for Studies on the Prevention of Alzheimer’s Disease, Douglas Mental Health University Institute, Montreal, Quebec, Canada; b Department of Psychiatry, McGill University, Montreal, Quebec, Canada; c McGill Centre for Integrative Neuroscience, McGill University, Montreal, Quebec, Canada; d Department of Neurology and Neurosurgery, McGill University, Montreal, Quebec, Canada; e McConnell Brain Imaging Centre, Montreal Neurological Institute, Montreal, Quebec, Canada; f Division of Psychiatry, Faculty of Brain Sciences, University College London, London, United Kingdom

**Keywords:** Alzheimer’s disease, Amyloid, Cognition, Mindfulness, Prevention, Tau

## Abstract

**Background:**

Mindfulness, defined as nonjudgmental awareness of the present moment, has been associated with an array of mental and physical health benefits. Mindfulness may also represent a protective factor for Alzheimer’s disease (AD). Here, we tested the potential protective effect of trait mindfulness on cognitive decline and AD pathology in older adults at risk for AD dementia.

**Methods:**

Measures of trait mindfulness, longitudinal cognitive assessments, and amyloid-β (Aβ) and tau positron emission tomography scans were collected in 261 nondemented older adults with a family history of AD dementia from the PREVENT-AD (Pre-symptomatic Evaluation of Experimental or Novel Treatments for AD) observational cohort study. Multivariate partial least squares analyses were used to examine relationships between combinations of different facets of trait mindfulness and 1) cognitive decline, 2) Aβ, and 3) tau.

**Results:**

Higher levels of mindful nonjudgment, describing, and nonreactivity were associated with less cognitive decline in attention, global cognition, and immediate and delayed memory. Higher levels of mindful nonjudgment and nonreactivity were related to less Aβ positron emission tomography signal in bilateral medial and lateral temporoparietal and frontal regions. Higher levels of mindful acting with awareness, describing, nonjudgment, and nonreactivity were associated with less tau positron emission tomography signal in bilateral medial and lateral temporal regions.

**Conclusions:**

Trait mindfulness was associated with less cognitive decline and less Aβ and tau in the brain in older adults at risk for AD dementia. Longitudinal studies examining the temporal relationship between trait mindfulness and AD markers, along with mindfulness intervention studies, will be important for further clarifying the potential protective benefits of mindfulness on AD risk.

Mindfulness is defined as the ability to engage in nonjudgmental awareness of the present moment (1). Growing evidence suggests that both the dispositional trait and the formal practice of mindfulness are associated with an array of mental and physical health benefits, including reduced depression, anxiety, and stress (2); improved sleep (3); less pain (4); and better cardiovascular function (5). Mindfulness has also been related to changes in brain structure and function (6), along with better cognitive performance (7), including in older adults (8). The goal of the current study was to examine the possible protective benefits of trait mindfulness on cognitive decline and Alzheimer’s disease (AD) pathology in nondemented older adults at risk of AD dementia.

In the absence of an imminent treatment or cure for AD dementia, increasing focus is being placed on early prevention. Encouragingly, up to 40% of the risk for dementia is attributed to modifiable behavioral factors (9). It is estimated that even a 10% reduction in these risk factors would reduce the prevalence of AD-related dementia by over 8% (10). AD is characterized by a long preclinical stage, involving the accumulation of amyloid-β (Aβ) and tau pathology, along with subtle cognitive decline, up to two decades prior to the onset of dementia (11). This phase offers a window of opportunity for studying the behavioral factors associated with the earliest stage of AD.

Leveraging a longitudinal cohort sample, we tested the potential protective effect of trait mindfulness on cognitive decline in older adults at risk of AD dementia. We also assessed the association between trait mindfulness and AD pathology, with the hypothesis that mindfulness could reduce dementia risk by providing resistance against AD pathology (12). That is, individuals who are more mindful may have low levels of Aβ and tau despite being at risk for AD. We collected measures of trait mindfulness along with longitudinal cognitive assessments and Aβ and tau positron emission tomography (PET) scans in older adults at increased risk of AD. We then used multivariate statistical analyses to examine associations between combinations of different aspects of trait mindfulness and 1) cognitive decline, 2) Aβ, and 3) tau.

## Methods and Materials

### Participants and Study Design

A total of 261 participants were included in this study from the PREVENT-AD (Pre-symptomatic Evaluation of Experimental or Novel Treatments for AD) cohort. This ongoing longitudinal observational study enrolls older adults who are cognitively normal but have a parent or at least 2 siblings diagnosed with AD-like dementia, placing them at increased risk for sporadic AD (13). Other enrollment criteria include being over 60 years of age (or between 55 and 59 years of age if their age was <15 years from their parent’s age of symptom onset), an absence of major neurological and psychiatric diseases, and intact cognition based on the Montreal Cognitive Assessment (score above 25) (14) and the Repeatable Battery for the Assessment of Neuropsychological Status (RBANS) (15) at the time of enrollment. Study enrollment spanned from 2012 to 2017, while longitudinal data collection, including annual cognitive assessment with the RBANS, is ongoing. Since 2017, additional measures have been added to the study. In 2017 to 2019, a subset of participants underwent Aβ and tau PET scans at a single time point, and in 2018, a measure of trait mindfulness was collected (with an additional 16 participants completing it in 2020–2021). All participants included in the current analyses completed the measure of trait mindfulness and at least one annual cognitive assessment (*N* = 261), with 124 of these individuals also completing Aβ and tau PET scans. Further details of the relative timing of measures are contained within Supplemental Methods. Participants were cognitively normal at the time of their baseline cognitive assessment and PET scans, though 12 individuals (including 5 PET participants) developed mild cognitive impairment (MCI) prior to completion of the mindfulness questionnaire. Results remain predominantly similar when these participants with MCI are excluded from analyses (see Supplemental Results).

### Mindfulness

The 39-item self-report Five Facet Mindfulness Questionnaire (FFMQ) (16) was collected for all participants (*N* = 261). The questionnaire was administered via an online platform for most participants, though 10 individuals chose to receive a paper copy in the post and a further 16 participants completed it over the telephone. The FFMQ contains five subscales measuring different facets of trait mindfulness: observing (being aware of, and paying attention to, internal and external stimuli and experiences), describing (verbally labeling and expressing one’s experiences), acting with awareness (paying conscious attention to what is happening in the present moment, rather than acting automatically), nonjudgment (not judging one’s own experiences), and nonreactivity (allowing experiences to arise without reacting to them). Participants were instructed to rate each item based on the extent to which the statements applied to them, on a Likert scale of 1 (never or very rarely true) to 5 (very often or always true). Scores were calculated for each mindfulness subscale, with higher scores reflecting higher levels of mindfulness. The nonreactivity subscale consists of seven items, whereas the remaining subscales consist of eight items. Scores for nonreactivity therefore ranged from 7 to 35, while the remaining subscale scores ranged from 8 to 40. Missing values were replaced with the mean of the subject’s responses for each subscale. Three participants with >10% of missing values were excluded from analyses. The FFMQ has demonstrated reliability and validity in clinical as well as in nonclinical populations (16,17).

### Cognition

As part of the PREVENT-AD study, participants underwent annual cognitive testing using the RBANS. The RBANS comprises 12 cognitive subtests, which produce five cognitive domain index scores (immediate memory, attention, visuospatial construction, language, and delayed memory), along with a total score reflective of global cognition. Scores are standardized to a mean score of 100 with an SD of 15. Longitudinal cognitive assessment (≥2 time points) was available for 257 (98.5%) participants, with a median follow-up time of 5 years (range, 1–7 years). The FFMQ was collected a mean of 1429.29 days (i.e., ∼3.9 years) after the baseline cognitive assessment (SD = 620.97 days). For further details of the closest cognitive time point to the FFMQ for each participant, see the Supplemental Methods.

### Neuroimaging Measures

#### Image Acquisition

A subset of participants (*n* = 124) underwent PET imaging using [^18^F]NAV4694 to assess Aβ burden and [^18^F]AV1451 (flortaucipir) for tau deposition. Aβ and tau PET scans were preprocessed using a standard pipeline (https://github.com/villeneuvelab/vlpp) (see Supplemental Methods). Standardized uptake value ratios (SUVRs) for each of the Desikan-Killiany regions were calculated using reference regions of the whole cerebellum gray matter for Aβ PET scans (18) and the inferior cerebellar gray matter for tau PET (19). SUVRs from regions demonstrated to accumulate Aβ and tau early in the disease course were included in subsequent analyses. For Aβ, this included 38 SUVRs from the 19 bilateral regions that form the global Aβ index, namely the lateral and medial prefrontal, parietal, lateral temporal, and cingulate cortices (20). For tau, 14 SUVRs were included, comprising 7 bilateral regions accumulating early tau pathology in preclinical AD (21). This included regions from Braak stages I (entorhinal cortex) and III (amygdala, and fusiform, parahippocampal, and lingual gyri) and temporal regions from Braak stage IV (inferior and middle temporal gyri). Braak stage II (i.e., the hippocampus) was excluded due to off-target binding in the adjacent choroid plexus (22).

### Statistical Analysis

#### Primary Analyses

In order to explore the relationships between combinations of mindfulness facets and 1) longitudinal cognition, 2) Aβ, and 3) tau, three separate multivariate partial least squares (PLS) analyses were employed (23) using PLS Software v6.15.1 on MATLAB v2018a (The MathWorks, Inc.). PLS takes two sets of variables (from two matrices) and uncovers linear combinations of these variables that maximally correlate with one another (24). For all three PLS analyses, the first matrix contained each mindfulness facet (i.e., observing, describing, acting with awareness, nonjudgment, and nonreactivity) in a separate column, with rows representing individual participants. Mindfulness data were *z*-scored due to the differing ranges of each subscale. The second matrix contained separate columns for each of the dependent variables, namely each of the six cognitive change scores for PLS 1 (calculated using linear mixed-effects models, see Supplemental Methods) or PET SUVRs for each region of interest for PLS 2 and 3 (38 regions for Aβ, 14 regions for tau). Each row represented a single participant.

The PLS analyses produced sets of latent variables relating the mindfulness facets with each dependent variable (longitudinal cognition, Aβ, and tau). Each latent variable consisted of a singular value, a vector of weights for each mindfulness facet, and a vector of weights for each dependent variable. Permutation tests were used to identify the significant latent variables (*p* < .05). Then, for each significant latent variable, bootstrap resampling was employed to ascertain the most stable mindfulness subscales and cognitive scores/brain regions contributing to the multivariate relationship. For the mindfulness subscales, the SE of the resampled distribution was calculated. The mindfulness facets for which the SE values did not overlap with zero were considered as significantly contributing to the multivariate relationship with the dependent variable. A bootstrap ratio was produced for each of the dependent variables. Cognitive scores/brain regions with bootstrap values >1.96 were considered as significant contributors to the multivariate relationship with mindfulness. For Aβ and tau PET PLS analyses, bootstrap ratios for each of the Desikan-Killiany regions of interest were projected on 2-dimensional brain representations using the ggseg package v1.6.01 (25) in R v1.1.463 (R Foundation for Statistical Computing). Finally, the vectors of weights for each mindfulness subscale and dependent variable were multiplied by the original data for these variables for each participant, producing a weighted score of mindfulness facets and a weighted score of longitudinal cognition, Aβ, and tau for each participant. These scores were then used in subsequent linear regression analyses to examine the potential effect of covariates on the multivariate relationship between mindfulness and the dependent variables. In these models, implemented using the lm function in R, the effect of the weighted mindfulness score on predicting the weighted dependent variable score was explored while controlling for age at the time of FFMQ completion, sex, years of education, *APOE* status (ɛ4 carrier vs. noncarrier), and number of days between completion of the FFMQ and the baseline cognitive assessment/PET scans.

#### Complementary Analyses

Given that we have previously documented relationships in the PREVENT-AD dataset between psychological variables, such as personality and neuropsychiatric factors, and longitudinal cognition, Aβ, and tau (24,26), we were also interested in examining the potential influence of these other psychological factors on the multivariate relationships between mindfulness and longitudinal cognition, Aβ, and tau. Participants completed questionnaire measures of personality traits (i.e., extraversion, agreeableness, conscientiousness, neuroticism, and openness) and neuropsychiatric symptoms (i.e., depression, anxiety, and stress) (see Supplemental Methods for further details). We first performed univariate Pearson correlation analyses to examine associations between mindfulness traits and the other psychological factors. Subsequently, we repeated our main PLS analyses between mindfulness and longitudinal cognition, Aβ, and tau, including these additional psychological variables alongside the mindfulness facets in the first PLS matrix.

### Standard Protocol Approvals, Registrations, and Patient Consents

All participants provided written informed consent, and all research procedures were approved by the Institutional Review Board at McGill University and complied with the ethical principles of the Declaration of Helsinki.

### Data Availability

Data may be made available by request by a qualified investigator.

## Results

Participant characteristics are displayed in Table 1. The PET subsample was representative of the whole cognition sample, except that the PET subsample was slightly older (*p* = .02). No differences in trait mindfulness were apparent between the *APOE* ɛ4 carriers versus noncarriers in either the full sample or the PET subsample (all *p* values >.08).

**Table 1:** Demographic, Pathological, and Clinical Characteristics of the Sample

	Cognition Sample, *N* = 261	PET Subsample, *n* = 124
Demographics		
Age, years, mean (SD)	67.27 (5.24)	68.60 (4.90)
Sex, female:male, *n* (% female)	186:75 (71.3%)	92:32 (74.2%)
Education, years, mean (SD)	15.50 (3.46)	15.25 (3.29)
*APOE* ɛ4 carriers, *n* (%)	101 (38.7%)	48 (38.7%)
MCI at time of FFMQ, *n* (%)	12 (4.6%)	5 (4.03%)
PET, Mean (SD)		
Global Aβ SUVR	–	1.34 (0.34)
Entorhinal tau SUVR	–	1.07 (0.14)
Cognition, Mean (SD)		
MoCA, baseline (out of 30)	28.11 (1.59)	28.16 (1.50)
RBANS, baseline		
Global cognition	102.82 (10.32)	102.73 (10.66)
Attention	107.02 (15.08)	106.42 (15.41)
Visuospatial	96.39 (13.63)	96.71 (13.55)
Language	103.75 (9.96)	104.02 (10.55)
Immediate memory	102.57 (11.39)	101.91 (10.67)
Delayed memory	102.33 (9.09)	102.48 (9.28)
Mindfulness (FFMQ), Mean (SD)		
Observing	27.55 (6.05)	28.31 (5.94)
Describing	29.51 (6.04)	30.28 (5.56)
Acting with awareness	31.01 (5.71)	31.23 (5.59)
Nonjudgment	30.66 (5.51)	31.03 (5.53)
Nonreactivity	23.11 (4.75)	23.63 (4.49)

Age was calculated at the time of FFMQ completion. *APOE* ɛ4 carriers had at least one copy of the ɛ4 allele.

Aβ, amyloid-β; FFMQ, Five Factor Mindfulness Questionnaire; MCI, mild cognitive impairment; MoCA, Montreal Cognitive Assessment; PET, positron emission tomography; RBANS, Repeatable Battery for the Assessment of Neuropsychological Status; SUVR, standardized uptake value ratio.

### PLS Analyses

#### Longitudinal Cognition

**Figure 1:**
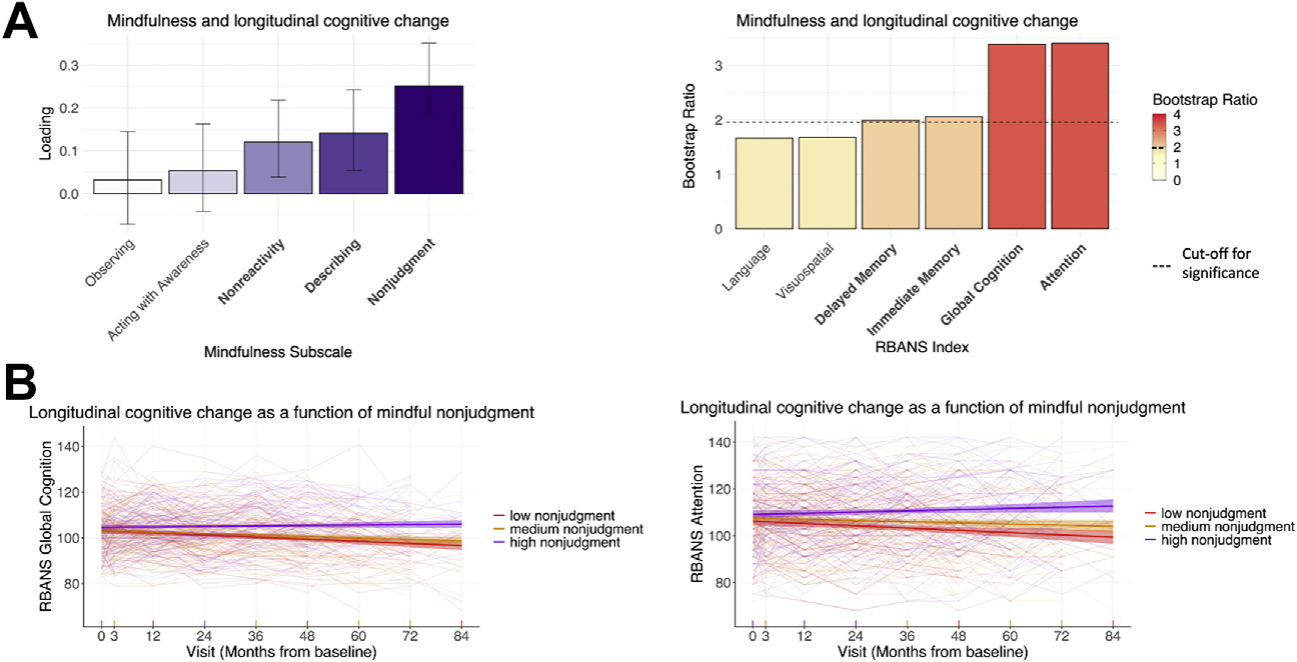
**(A)** Results of partial least squares analyses between mindfulness facets and longitudinal cognitive change. Significant mindfulness facets and cognitive indexes are presented in bold; significant cognition bootstrap ratios are also represented by cognitive indexes with colors above the dotted line on the color bar. **(B)** Longitudinal cognitive change for the most robust mindfulness facet and cognitive domains from the partial least squares analysis, with participants grouped into tertiles of high, medium, and low mindfulness for visualization purposes (solid lines; spaghetti plots represent individual trajectories). RBANS, Repeatable Battery for the Assessment of Neuropsychological Status.

For the PLS analysis between mindfulness and longitudinal cognition, two significant latent variables emerged (*p* < .001, *p* = .004), explaining 68.03% and 20.32% of the PLS variance, respectively. We report the first latent variable here, given that it explained the majority of the PLS variance and was replicated in the subset of 124 participants who completed Aβ and tau PET scans (Figure S1). Weights and standard errors for the mindfulness facets and bootstrap ratios for the cognitive index slopes for this latent variable are shown in Figure 1. Higher levels of mindful nonjudgment, describing, and nonreactivity were related to less cognitive decline in attention, global cognition, immediate memory, and delayed memory (Figure 1A). The correlation between the weighted mindfulness and longitudinal cognitive scores was 0.24 (*p* < .001), which accounted for 5.4% of the variance in cognitive change. This relationship between mindfulness and longitudinal cognitive decline remained significant when covariates (age, sex, education, *APOE* status, and time between completion of the FFMQ and baseline RBANS) were included in the model (β = 0.21, *t* = 3.55, *p* < .001). The main results from this PLS analysis were also replicated when participants who had MCI at the time of the FFMQ were removed from analyses, with the exception of the immediate and delayed memory scores, which did not reach statistical significance (Figure S2).

Figure 1B displays longitudinal cognitive change as a function of mindfulness scores, for the most robust cognitive and mindfulness subscales that contributed to the first latent variable (i.e., longitudinal attention and global cognition as a function of mindful nonjudgment). Participants are grouped into tertiles of high, medium, and low mindfulness for visualization purposes.

#### Amyloid-β

**Figure 2:**
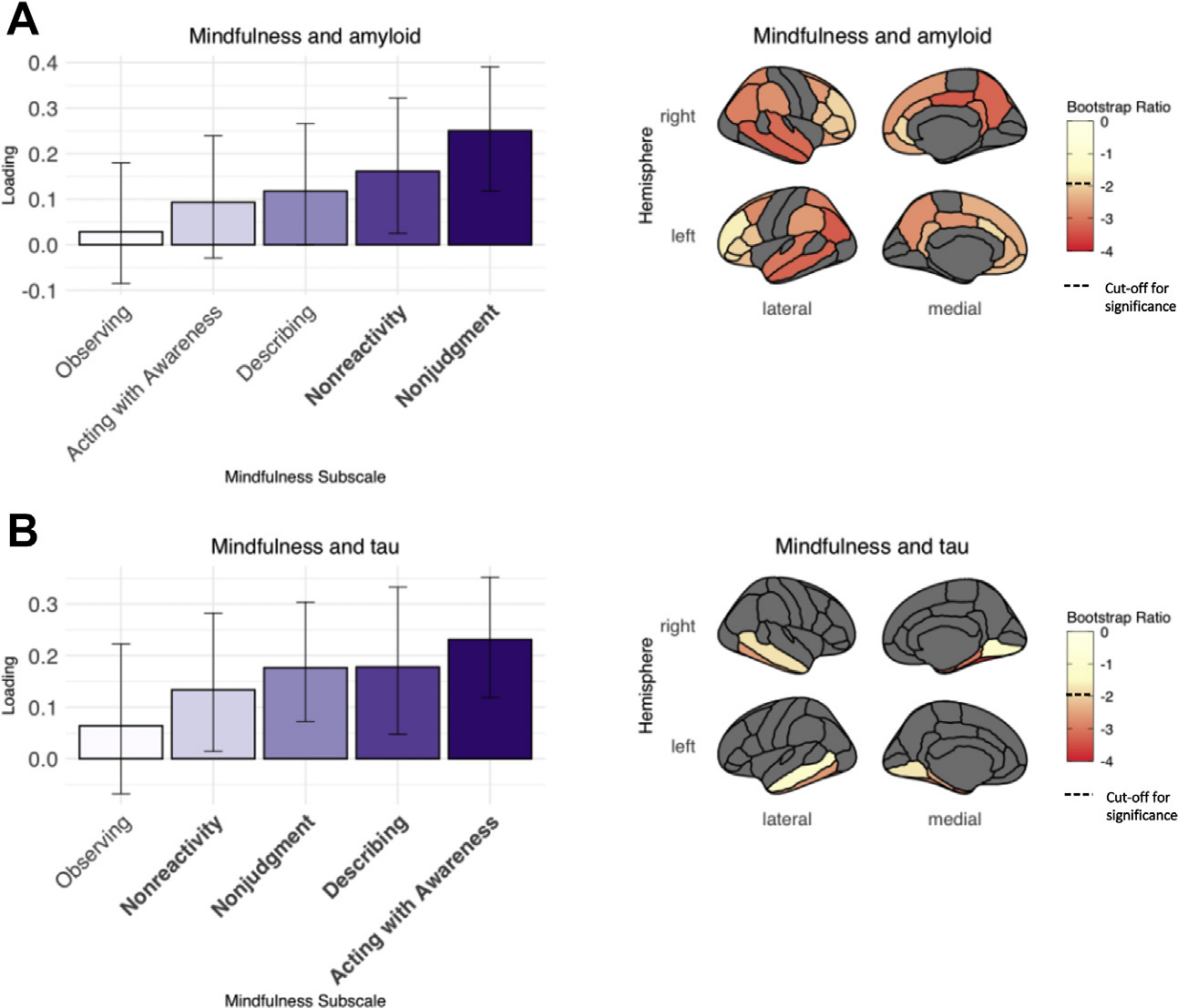
Results of partial least squares analyses between mindfulness traits and **(A)** amyloid-β and **(B)** tau standardized uptake value ratios. Error bars on bar plots represent the SEs. Significant mindfulness facets are presented in bold; significant amyloid-β and tau bootstrap ratios are represented by regions with colors below the dotted line on the color bars. Brain regions shown in gray were not included in analyses.

One significant latent variable emerged for the PLS analysis between mindfulness and Aβ (*p* = .005), which explained 97.72% of the PLS variance. Loadings and SEs for the mindfulness facets and bootstrap ratios for the Aβ regional SUVRs that comprise this latent variable are displayed in Figure 2A. Higher levels of mindful nonjudgment and nonreactivity were associated with lower Aβ burden in the bilateral medial and lateral temporoparietal and frontal regions (loadings for each brain region of interest are contained within Table S1). The correlation between the weighted mindfulness scores and the weighted Aβ regional values was 0.25 (*p* = .005), accounting for 5.4% of the variance in Aβ. The relationship between mindfulness and Aβ remained significant when covariates (age, sex, education, *APOE* status, and time between completion of the FFMQ and PET scans) were included in the model (β = 0.21, *t* = 2.34, *p* = .02). When participants with MCI at the time of the FFMQ were removed, PLS results remained predominantly similar, though the mindful nonreactivity subscale was no longer significant (Figure S3A).

#### Tau

For the PLS analysis between mindfulness and tau, one significant latent variable emerged (*p* = .007), explaining 92.58% of the PLS variance. Figure 2B shows the loadings and SE values for the mindfulness facets and bootstrap ratios for the regional tau SUVRs comprising this latent variable. Higher levels of mindful acting with awareness, describing, nonjudgment, and nonreactivity were related to a lower burden of tau in bilateral temporal regions, namely the entorhinal cortex; amygdala; and parahippocampal, fusiform, and inferior temporal gyri (see Table S1 for the loadings of each region of interest). The correlation between the weighted mindfulness scores and weighted regional tau SUVRs was 0.26 (*p* = .003), accounting for 6.1% of the tau variance. This relationship between mindfulness and tau remained significant when covariates (age, sex, education, *APOE* status, and time between completion of the FFMQ and PET scans) were included in the model (β = 0.25, *t* = 2.88, *p* = .005). Results of this PLS analysis remained unchanged when participants with MCI at the time of the FFMQ were excluded (Figure S3B).

### Complementary Analyses

**Figure 3:**
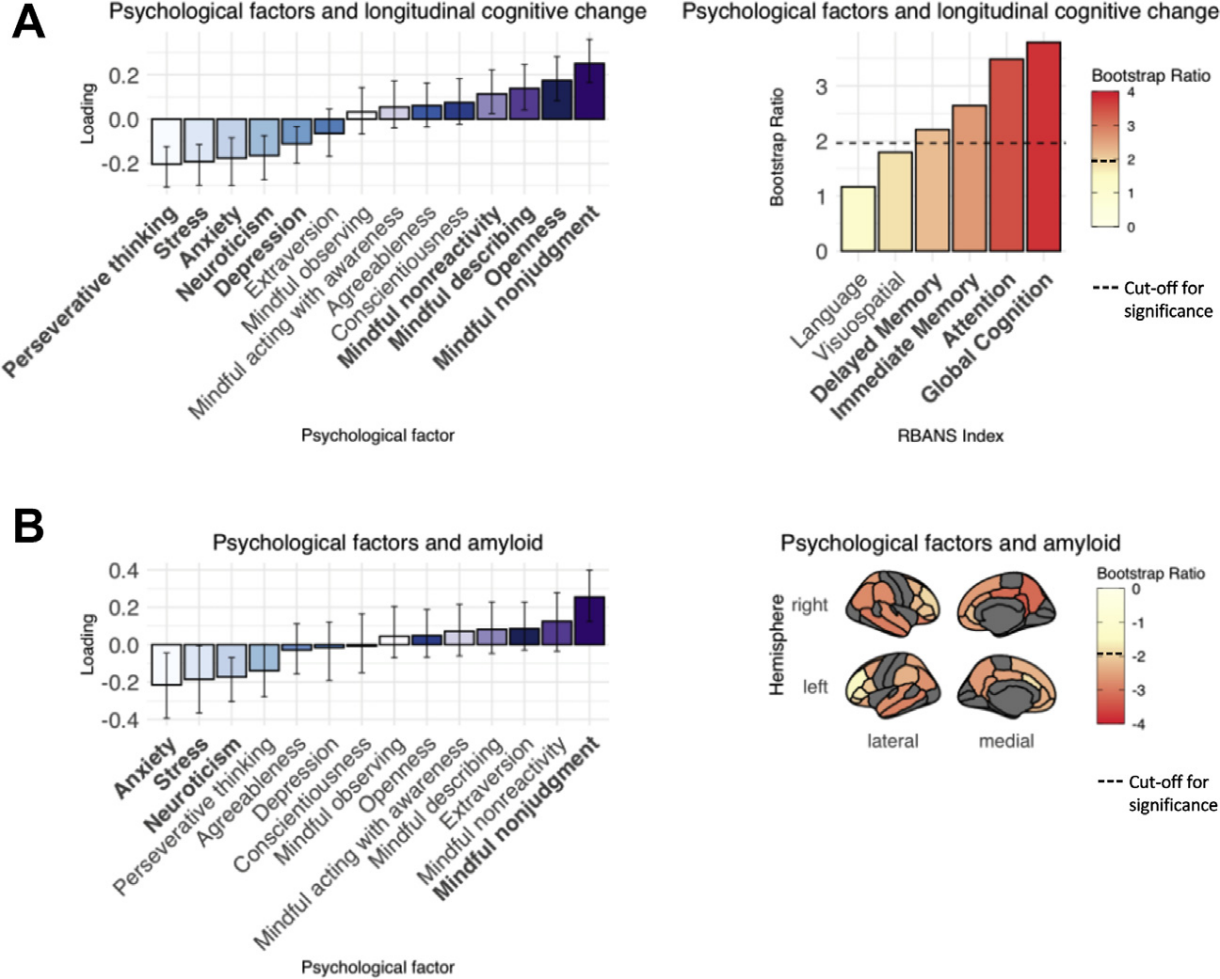
Results of partial least squares analyses between psychological variables and **(A)** longitudinal cognitive change and **(B)** amyloid-β standardized uptake value ratios. Error bars on bar plots represent the SEs. Mindfulness variables are presented in shades of purple, with the remaining psychological factors represented in shades of blue. **(A)** Significant psychological variables and cognitive indexes are presented in bold; significant cognition bootstrap ratios are also represented by cognitive indexes with colors above the dotted line on the color bars. **(B)** Significant psychological variables are presented in bold; significant amyloid-β bootstrap ratios are represented by regions with colors below the dotted line on the color bars. Brain regions shown in gray were not included in analyses. RBANS, Repeatable Battery for the Assessment of Neuropsychological Status.

Univariate correlation analyses between mindfulness and other psychological variables revealed that higher levels of mindfulness traits were correlated with less neuroticism, perseverative thinking, anxiety, stress, and depression, and with higher conscientiousness, openness, agreeableness, and extraversion (Figure S4). When the main PLS analyses between mindfulness and longitudinal cognition, Aβ, and tau were repeated including these additional psychological variables alongside the mindfulness facets, the majority of the previously uncovered relationships between mindfulness traits and AD markers remained significant, with the exception of the mindful nonreactivity subscale in both the Aβ and tau PLS analyses, which did not reach statistical significance (Supplemental Results; Figure S5). This suggests that the relationships between mindfulness and longitudinal cognition, Aβ, and tau are at least partially independent of other psychological factors. For Aβ, the addition of other psychological variables to the PLS analysis also did not explain any additional variance in Aβ compared with the mindfulness traits alone. Importantly, for both the longitudinal cognition (Figure 3A) and Aβ (Figure 3B) PLS analyses, mindful nonjudgment was the strongest contributing psychological variable to the multivariate relationship.

## Discussion

The potential health benefits of mindfulness have become increasingly recognized in recent years. In particular, mindfulness is posited to have positive effects on brain health, including improved cognition (7). Focusing on the preclinical stage of AD, here we explored multivariate relationships between trait mindfulness, cognitive decline, and AD pathology. In older adults at risk for sporadic AD dementia, higher levels of trait mindfulness were related to less cognitive decline and to less Aβ and tau deposition in the brain. Distinct combinations of mindfulness traits were related to cognitive decline, Aβ, and tau; however, mindful nonjudgment emerged as a common correlate of all three AD markers. Importantly, while mindfulness was related to other psychological variables including neuropsychiatric symptoms and personality traits, the main associations between mindfulness and cognitive decline, Aβ, and tau remained significant when these other psychological factors were included in analyses. Furthermore, when neuropsychiatric symptoms, personality traits, and mindfulness were all entered into the same model, mindful nonjudgment still showed the strongest association with Aβ and cognitive decline. Together, these results highlight a potential role for trait mindfulness as a psychological protective factor for AD.

In older adults at risk of AD dementia, higher levels of trait mindful nonjudgment, describing, and nonreactivity were related to less cognitive decline in global cognition, attention, and immediate and delayed memory over an average of 5 years. The associations between higher levels of mindfulness and less decline in global cognition and attention were also apparent in the subset (*n* = 249 of 261) of cognitively unimpaired participants. Mindfulness has previously been associated with better cognitive performance in older adults, particularly in domains of attention and executive function (8). By definition, attentional control is a primary component of mindfulness, given that it involves the directing of attention to one’s present-moment experience (1), with improvements in attentional control observed following mindfulness training programs, including in older individuals (27). Our findings suggest that increased levels of trait mindfulness may also be associated with the maintenance of attentional function over time in cognitively normal older adults at risk for AD dementia, along with other cognitive domains known to be affected in preclinical AD (28). Further longitudinal studies are required to determine whether older adults who are more mindful in daily life may be able to withstand cognitive decline associated with aging and/or AD, and indeed whether these associations translate to a delaying, or even prevention, of the onset of dementia.

In order to explore whether mindfulness could potentially influence dementia risk via providing resistance against AD pathology, we also examined associations between trait mindfulness and Aβ and tau deposition in the brain. In support of this hypothesis, higher levels of mindful nonjudgment and nonreactivity were related to less Aβ in the bilateral medial and lateral temporoparietal and frontal regions, including the precuneus, posterior cingulate cortex, and orbitofrontal cortex. These regions are among the first cortical areas to accumulate Aβ pathology early in the disease course (20,29,30). It is interesting to note that many of the Aβ regions associated with mindfulness belong to the default mode network, a widespread functional brain network known to be disrupted in early AD (31). Mindfulness has also been associated with changes in the activity and functional connectivity of the default mode network, particularly the posterior cingulate (32,33), which has been proposed as a key candidate mechanism for the effects of mindfulness on psychological well-being (34). Default mode network connectivity therefore represents a potential pathway by which mindfulness could be associated with Aβ pathology, which is worthy of further inquiry.

Higher levels of mindful acting with awareness, describing, nonjudgment, and nonreactivity were also related to less tau in regions known to accumulate pathology early in the course of AD, namely the entorhinal cortex; amygdala; and parahippocampal, inferior temporal, and fusiform gyri. Medial temporal lobe regions are believed to play important roles in mindfulness, with both the trait and formal practice of mindfulness associated with gray matter volume of the amygdala (35) and hippocampus (36), along with the functional connectivity profiles of these regions (37,38). Taken together, the associations uncovered here between mindfulness and Aβ and tau pathology in specific neural regions suggest that future studies examining the potentially mediating role of brain structure and function on these relationships may be of interest.

While distinct combinations of mindfulness traits were associated with cognitive decline, Aβ, and tau, mindful nonjudgment was the only subscale related to all three markers. The associations between mindful nonjudgment, cognition, and AD pathology were highly robust: remaining significant even with the exclusion of participants with MCI and the inclusion of other psychological variables in the analyses. Broadly, the construct of mindfulness is considered to incorporate both the attention to present-moment experiences and the attitude of acceptance toward these experiences (39). The mindful nonjudgment subscale of the FFMQ captures this latter component, namely the tendency to refrain from judging one’s own thoughts and feelings as they arise, via items such as “I judge my thoughts as good or bad” and “I criticize myself for having irrational emotions and thoughts” (both reverse-scored). Mindful nonjudgment has been proposed to play a critical role in reducing stress (40), alleviating symptoms of psychopathology (41), and improving cardiovascular health (42), over and above present-moment awareness. Our findings suggest that mindful nonjudgment may also be particularly associated with AD markers.

Preliminary work has begun to explore the effect of mindfulness-based interventions in MCI and early-stage AD dementia. The evidence is mixed regarding their effect on cognition, however, with one study reporting significant cognitive improvements in participants with MCI following an 8-week mindfulness intervention (43), though only trend-level differences from pre- to postintervention were observed in another (44). While these initial findings are promising, there remains a need for large, well-controlled studies of mindfulness interventions in early AD, with longer follow-up periods [see (45)]. These interventions may also be most effective if targeted at earlier, preclinical disease stages [e.g., (46, 47, 48)] prior to the onset of extensive and likely irreversible AD pathology, neural damage, and cognitive impairment (11). Results from the current study point to an association between trait mindfulness and markers of AD in the preclinical stage. Whether mindfulness interventions in this early disease stage may lead to slower rates of AD pathology accumulation and cognitive decline will form a key question for future research. Promisingly, self-reported mindfulness, such as that measured by the FFMQ, has been found to increase following mindfulness intervention programs (49), including in participants with MCI (43), suggesting that this trait may be modifiable. The specificity of such changes to mindfulness interventions, however, remains debated (49,50), which has contributed to some concerns about the validity of self-report measures in adequately capturing the construct of mindfulness (51,52). While the FFMQ has demonstrated construct validity (53) and is recommended as the most comprehensive self-report mindfulness tool for use in the general population (52), validating the current findings using experimental manipulations of mindfulness (particularly nonjudgment), and employing appropriate control groups, will be particularly important for future studies.

There are numerous potential psychological, behavioral, and physiological mechanisms by which mindfulness may be associated with AD pathology and cognitive decline. For example, positive impacts of mindfulness on reducing depression, anxiety, and stress (2) and modifying personality traits such as neuroticism (54) have been observed. These psychological factors are also associated with increased risk for AD dementia (55,56) and higher levels of AD pathology and cognitive decline in preclinical disease stages (24,57,58). In the current study, mindfulness traits were associated with perseverative thinking, depression, anxiety, stress, and personality traits in the expected directions. Crucially, however, the main associations between mindfulness and cognition and mindfulness and AD pathology remained apparent even when these other psychological traits were included in analyses. In fact, for both the longitudinal cognition and Aβ analyses, mindful nonjudgment was the strongest contributing psychological variable to the multivariate relationship. Accordingly, the associations between mindfulness and AD markers are unlikely to be fully explained by its indirect effects on other psychological variables.

Other mechanisms are therefore likely involved in the relationships between mindfulness and AD markers. Mindfulness has been associated with an array of physical health benefits, involving similar pathways that are proposed to be involved in AD. Poor cardiovascular health is one of the most significant categories of risk factors for the development of AD pathology and its associated dementia syndrome (9,59,60). Mindfulness has also been linked to an array of benefits on vascular health, including blood pressure, heart rate variability, and glucose regulation (5). These effects may be at least partly attributable to the influence of mindfulness on positive health behaviors such as physical activity, smoking cessation, diet, and sleep (5,61). Additional physical health markers such as inflammation, physiological stress (including cortisol), immune system dynamics, and cellular aging have also been associated with both mindfulness (62, 63, 64, 65) and AD (66, 67, 68, 69). More research is required to further explore the relative contribution of each of these potential pathways to the association between mindfulness and early markers of AD.

Overall, higher levels of trait mindfulness were associated with better cognition over time and less Aβ and tau in the brain in older adults at risk for AD dementia. Our multivariate approach permitted in-depth profiling of associations between specific combinations of mindfulness facets and AD markers, though such relationships were also apparent using standard univariate analyses (see Table S2). Albeit not causal, the current findings raise the possibility that individuals who are more mindful may be more resistant to AD pathology and cognitive decline. By this view, cultivating mindfulness in daily life may be a way to reduce vulnerability to AD. As our study was correlational, however, and the participants were approaching the expected age of disease onset, it is also possible that lower mindfulness represents an early symptom of AD. Other limitations of our study include the cross-sectional nature of the PET scans, which were only conducted in a subset of participants. Accordingly, large-scale, longitudinal studies examining the temporal relationship between trait mindfulness and AD markers, along with mindfulness intervention studies with cognition and pathology as outcome variables, will be of utmost importance in refining our understanding of the potential protective benefits of mindfulness on resistance to AD pathology and cognitive decline.

## Supplementary Material

Supplementary Material

Key Resources Table
